# Two-stage palatal repair in non-syndromic CLP patients using anterior to posterior closure is associated with minimal need for secondary palatal surgery

**DOI:** 10.1186/s13005-024-00418-0

**Published:** 2024-03-09

**Authors:** Philipp Kauffmann, Johanna Kolle, Anja Quast, Susanne Wolfer, Boris Schminke, Philipp Meyer-Marcotty, Henning Schliephake

**Affiliations:** 1 Department of Oral and Maxillofacial Surgery, University Medical Center Goettingen, Goettingen, Germany; 2 Department of Orthodontics, University Medical Center Goettingen, Goettingen, Germany; 3 Georg-August-University Goettingen, Robert-Koch-Straße 40, Goettingen, 37099 Germany

## Abstract

**Objective:**

The aim of the present study was to assess the need for secondary palatal corrective surgery in a concept of palate repair that uses a protocol of anterior to posterior closure of primary palate, hard palate and soft palate.

**Methods:**

A data base of patients primarily operated between 2001 and 2021 at the Craniofacial and Cleft Care Center of the University Goettingen was evaluated. Cleft lips had been repaired using Tennison Randall and Veau-Cronin procedures in conjunction with alveolar cleft repair. Cleft palate repair in CLP patients was accomplished in two steps with repair of primary palate and hard palate first using vomer flaps at the age of 10–12 months and subsequent soft palate closure using Veau/two-flap procedures 3 months later. Isolated cleft palate repair was performed in a one-stage operation using Veau/two-flap procedures. Data on age, sex, type of cleft, date and type of surgery, occurrence and location of oronasal fistulae, date and type of secondary surgery performed for correction of oronasal fistula (ONF)and / or Velophyaryngeal Insufficiency (VPI) were extracted. The rate of skeletal corrective surgery was registered as a proxy for surgery induced facial growth disturbance.

**Results:**

In the 195 patients with non-syndromic complete CLP evaluated, a total number of 446 operations had been performed for repair of alveolar cleft and cleft palate repair (Veau I through IV). In 1 patient (0,5%), an ONF occurred requiring secondary repair. Moreover, secondary surgery for correction of VPI was required in 1 patient (0,5%) resulting in an overall rate of 1% of secondary palatal surgery. Skeletal corrective surgery was indicated in 6 patients (19,3%) with complete CLP in the age group of 15 – 22 years (*n* = 31).

**Conclusions:**

The presented data have shown that two-step sequential cleft palate closure of primary palate and hard palate first followed by soft palate closure has been associated with minimal rate of secondary corrective surgery for ONF and VPI at a relatively low need for surgical skeletal correction.

## Introduction

Palate closure in patients with cleft lip palate (CLP) continues to be subject to controversy since many decades. There is a plethora of different approaches with respect to surgical procedures and timing with little evidence of superiority of one protocol over another [1–4]. The aims of palatal closure, namely tight closure of the oronasal communication, sufficient length and mobility of the soft palate for adequate velopharyngeal function and minimal disturbance of maxillary growth are unquestioned. Key points of discussion in cleft palate surgery are one stage versus two stage repair, anterior versus posterior closure first and early or late closure of the hard palate, the latter being considered as major factor of influence for maxillary development [5]. However, the contribution of palatal surgery alone on maxillary growth and development of dental arches is difficult to assess in CLP patients. Surgery for lip closure has shown to have a major influence on maxillary development [6–8] and a well-coordinated adjuvant orthodontic treatment contributes substantially to the development of dental arches and the alveolar crest [9], leading to contradictory results when long-term effects of palatal surgery on maxillary growth were considered [10]. In contrast, fistula formation and velopharyngeal competence are direct sequelae of palatal surgery and the occurrence of oronasal fistulas and the subsequent need for secondary corrective surgery may be considered as a surrogate parameter for the ability of a treatment concept to achieve the surgical goals [11].

Cleft palate repair appears to be more prone to secondary surgical interventions than cleft lip repair. In a national survey, Thompson et al. reported a rate of lip revision surgery of 22.2 to 24.9% whereas palate revision surgery had shown a rate of 35.8 and 36.8% [12]. The occurrence of oronasal fistulae (ONF) contributes to this figure with a frequency of 4.9—8.6% reported in systematic reviews [13, 14]. The type of cleft appears to be significantly associated with the occurrence of ONF with a more than 3-fold higher incidence in CLP vs CP alone (17.9 vs. 5.4%) [14]. Secondary surgery due to velopharyngeal insufficiency (VPI) and speech problems in medium sized cohorts have been reported for 13.3 to 25.9% of the patients [15, 16]; a recent meta-analysis identified a 21% rate of additional surgical interventions for palatal re-repair for speech problems [17].

The effect of the sequence of individual surgical procedures on the rate of secondary corrections has been repeatedly discussed. However, a major multinational endeavor for comparison of four different strategies in a randomized approach has not been able to identify a preferable sequence of surgical interventions with respect to rate of fistula formation and velopharyngeal function [1, 2]. Early hard palate closure using a vomer flap during lip repair followed by soft palate repair has been associated with a low incidence of ONF [11, 18, 19] but the rate of secondary interventions for VPI with this approach has not been reduced [2].

The present study aims to contribute to this discussion with the results of a concept that copies the natural sequence of the embryological fusion process from anterior to posterior in three individual steps to minimize the need for secondary surgical procedures after primary cleft surgery in CLP patients.

## Material & methods

**Fig. 1:**
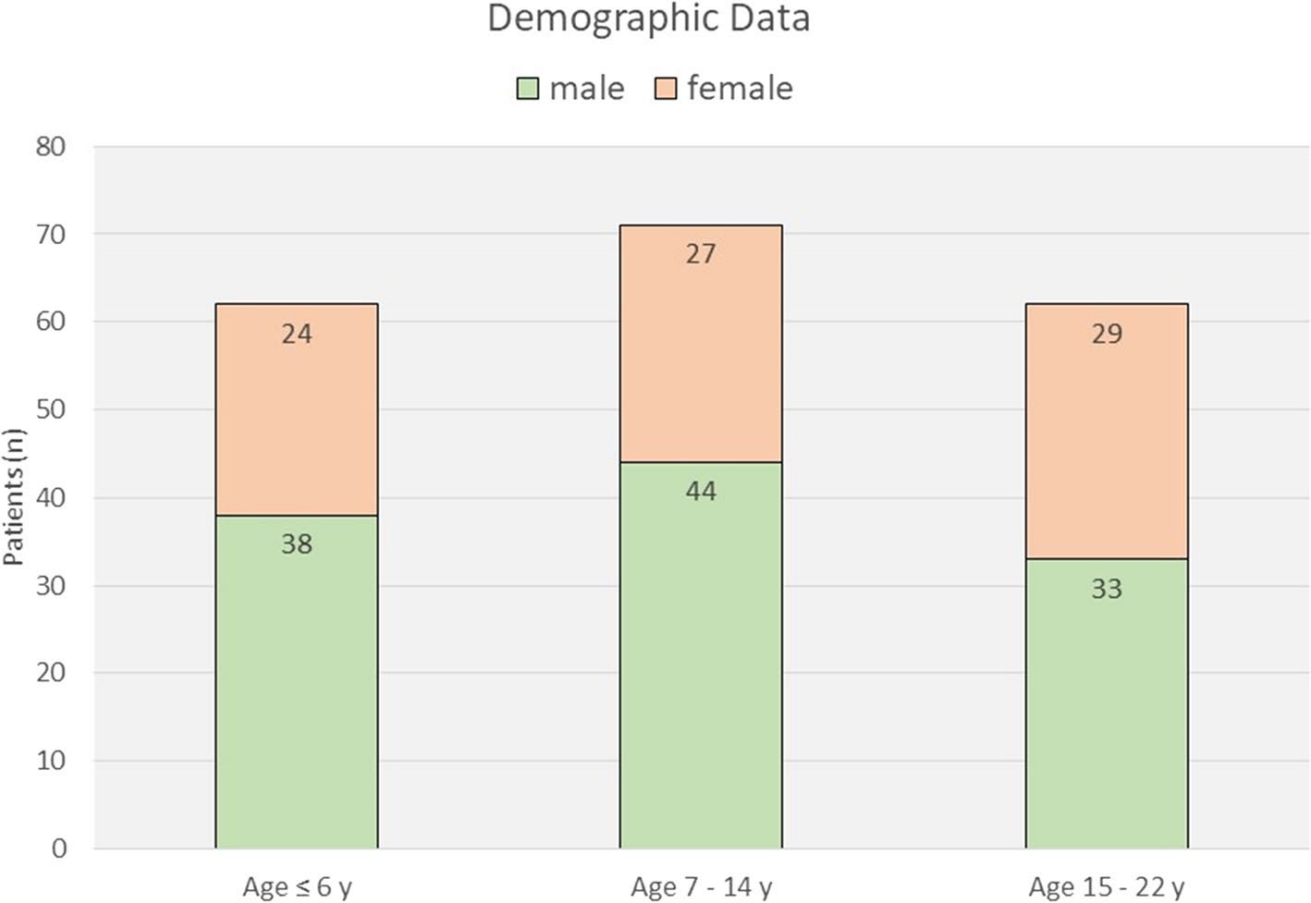
Demographic data

The study protocol had been approved by the local ethical committee (Registration Number 21/4/20). A cross-sectional analysis of patients listed in the data bank of the Craniofacial and Cleft Care Center (CCCC) at the University Medicine / Göttingen was performed. Data of all non-syndromic CLP patients were collected from the database (Access 2016, Microsoft Windows 10 Pro) who had been treated between August 2001 and December 2021 at the CCCC. The database contained data of 423 patients with cleft deformities of various nature, one hundred forty-nine of whom were “adopted” after treatments performed before August 2001 or previously performed elsewhere and 274 patients treated primarily and completely at the CCCC. Only data from the latter group of patients were evaluated listing date of birth, sex, type of cleft, date and type of surgery, occurrence and location of ONF, date and type of secondary surgery performed for correction of ONF or VPI as well as skeletal surgery at the end of growth. The type of cleft was categorized into cleft lip (CL), cleft lip alveolus (CLA), cleft lip palate (CLP). The cleft palate type was classified according to the system of Veau (Type I: soft palate, Type II: soft and hard palate, Type III: soft and hard palate and unilateral primary palate (UCLP), Type IV: soft and hard palate and bilateral primary palate (BCLP). Submucosal soft palatal clefts were classified as Veau I. A total of 195 patients with non-syndromic CLP and CP were selected for evaluation on the basis of this algorithm. Demographic data on distribution of gender and age are shown in Fig. 1.

### Surgical procedures

**Fig. 2:**
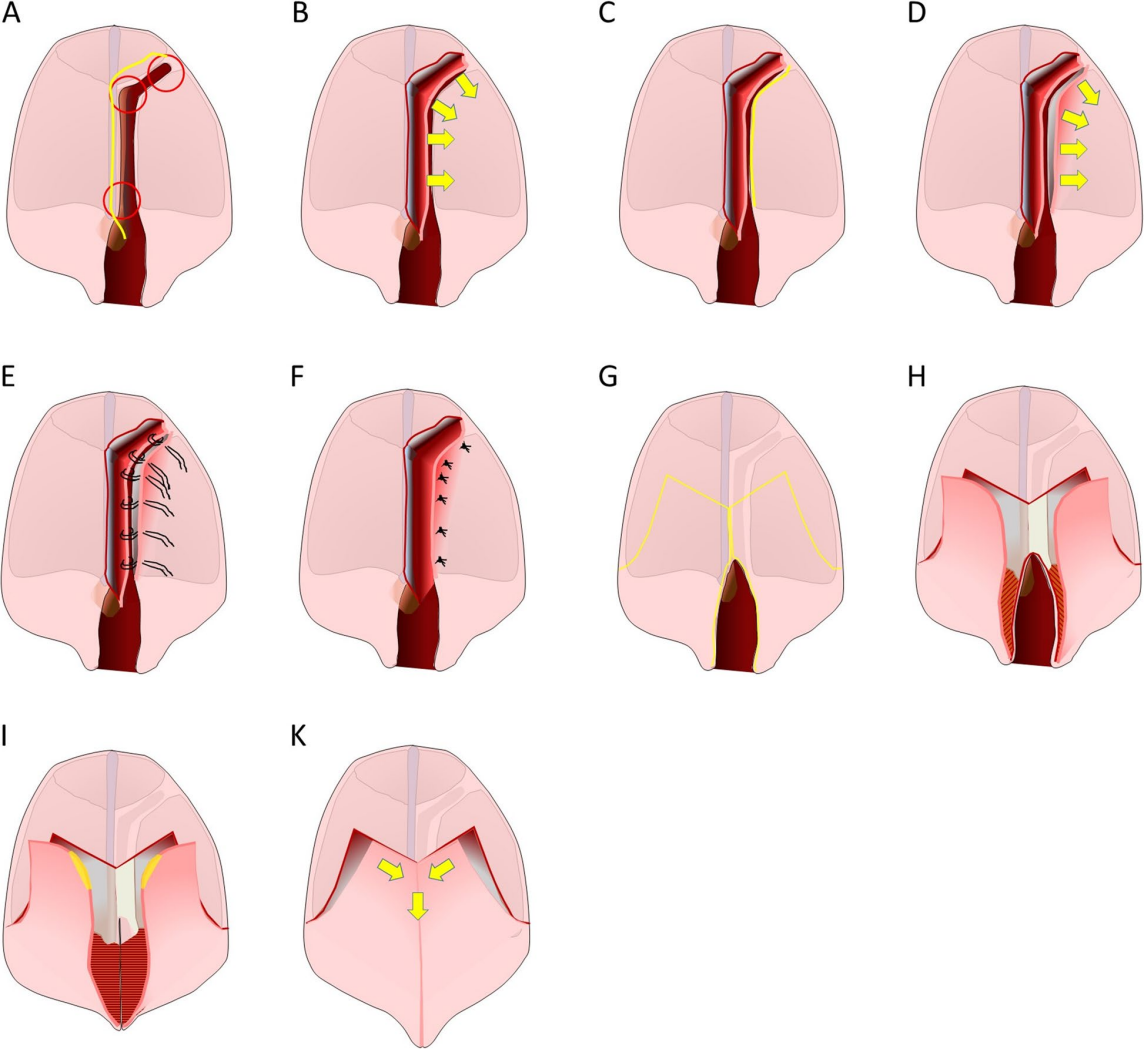
**A** Unilateral complete cleft lip and palate. Red circle identify the critical transition areas between cleft alveolus and primary palate, between primary and secondary palate and between hard and soft palate. Incision lines on the medial side of the cleft in yellow for the extended Vomer flap that includes the mucoperiosteum of the premaxilla and extends to the buccal side of the alveolar cleft. The buccal part of the alveolar cleft and anterior part of the nasal floor had been already closed during lip repair. **B** Mobilization of the mucoperiosteum of the premaxilla and the vomer towards the lateral edge of the cleft. Increased mobility is gained by subperiosteal dissection in cranial direction. **C** Incision line on the lateral side of the cleft extending to the buccal side of the closed alveolar cleft. **D** Mobilisation of the lateral mucoperiosteum of the palatal bone for at least 5 mm. **E** Preparation of back-and-forth sutures that unite the periosteal surfaces of the of the medial mucoperiosteum of the premaxilla / vomer with the lateral mucoperiosteum of the palatal bone. **F** Upon activation a safe overlap of at least 2–3 mm between the two periosteal surfaces should be achieved.** G** After 3 months, the residual cleft soft palate is addressed with a typical Veau incision (in yellow) extending at least 5 mm beyond the border between the soft and the hard palate, making sure, that enough mucoperiosteum overlying the hard palate is involved. **H** Elevation of the mucoperiosteum on the cleft side has to be done carefully with blunt preparation through the scar tissue of the former vomer flap bridging the ledge of the palatal bone and the vomer / contralateral palatal bone. **I** Release of the false insertion of the palatal muscles and reconstruction of the muscular sling is done in typical manner after mobilization and suturing of the nasal mucoperiosteal layer. Bilateral excision of small wedges (in yellow) of the palatal mucoperiosteum at the anterior medial flap egdes. **K** Medialization of the palatal flaps for reconstruction of the oral layer with simultaneous “push back” through the VY elongation

The presurgical treatment and the surgical protocol were identical for all patients with identical cleft types. Surgery was performed by the senior cleft surgeon of the team:

i) We start with a palate plate immediately after birth in CLP Patients and 2 weeks later with the attachment of the NAM appliances in complete CL, CLA and CLP cases.
ii) Cleft lip closure (unilateral: Tennison-Randall, bilateral: Veau-Cronin) with primary rhinoplasty and buccal mucosal advancement flap / periosteoplasty for closure of vestibular part of alveolar clefts (if applicable) at the age of 4 – 6 months. In very wide bilateral clefts, a two stage Veau-Cronin procedure was used. During lip closure, subperiosteal mobilization of the cleft sided buccal soft tissues of the maxilla up to the infraorbital rim with dissection of perinasal muscles were carried out to mobilize and reconstruct the “Delaire matrix” of facial soft tissues.
iii) In CLP patients, hard palate closure including primary palate repair using a Vomer flap at the age of 10 – 12 months was carried out. Care was taken to extend the incision on the vomer edge anteriorly into the mucoperiosteum of the premaxilla with an extended subperiosteal dissection in cranial direction to achieve sufficient mobility for a secure tension-free closure of the primary palate cleft and the palatal side of the alveolar cleft (Fig. 2A through F).
iv) Subsequently, soft palate closure was done 3 months later using a Veau / two-flap procedure with reconstruction of the palatal muscle system and push back of the palatal mucoperiosteal flaps at the age of 13 – 15 months. Care was taken to extend the incision for the anterior edge of the palatal flaps at least 5 mm anterior of the ledge of the hard palate to avoid ONF at the hard/soft palate border (Fig. 2G through K).

Isolated cleft palates were repaired in a single surgical procedure. Veau I clefts were closed using a Veau-two-flap procedure with reconstruction of the palatal muscle system and push back of the palatal flaps. In Veau II clefts, bilateral vomer flaps were elevated and sutured to the mobilized nasal mucosa to reconstruct the floor of the inferior nasal meatus in combination with a Veau / two-flap procedure as described above. In these clefts, the palatal flaps were extended as far anterior as possible.

No feeding tubes were used, breast feeding (if applicable) was allowed at the second postoperative day, otherwise nursing bottles were used.

### Follow-up

Patients were followed up once per year until the age of 18 by the whole multidisciplinary cleft team (OMF-surgeon, orthodontist, speech pathologist) during annual visits (month of birth). In case of skeletal corrective surgery, patients were followed until termination of postoperative orthodontic treatment (max. 22 years). A detailed analysis of speech quality was done at the age of three years, regular checks of speech quality were performed every second year. Speech therapy was provided near patients’ home outside of the CCCC.

Orthodontic therapy was provided routinely from the age of 6, in case of early skeletal discrepancies from the age of 4 with removable appliances. In case of deformation of the alveolar crest in conjunction with substantial malpositioning of teeth, fixed appliances were used in the permanent dentition. Orthodontic therapy was provided both at the CCCC and in the periphery closer to patients’ homes.

The occurrence of ONF was defined according to Muzaffar et al. as a failure of healing or a breakdown in the primary surgical repair of the palate [20]. However, we extended this definition to the surgical repair of the palatal part of the alveolar cleft and the primary palate, so that all oronasal communications that were visible or could be sounded in the area of the repaired alveolar cleft / primary cleft palate / incisor foramen and hard / soft palate cleft were registered as ONF. Surgery was indicated in case of fluid / food leakage or air loss during speaking. A need for corrective velopharyngeal surgery was indicated by the speech pathologist and the OMF surgeon during annual recalls when there was no acceptable increase in quality of speech achieved despite at least three years of continuous speech therapy.

Additionally, secondary skeletal corrective surgery was registered as a proxy to surgically induced growth retardation. The need for skeletal surgery was identified during the age of 16 – 18 years, when orthodontic therapy had not resulted in a Class I occlusion and radiological parameters indicated a cleft related skeletal malformation. Surgery was performed at the age between 17.8 and 18.5 years.

Patient data were analyzed for follow-up intervals of ≤6 years, 7 - 14 years, and 15-22 years.

### Statistical analysis

A Chi^2^ test was used to test for associations between cleft type and the location of ONFs as well as the need for secondary surgery. A *p*-value < 0.05 was considered as significant.

## Results

### Primary surgery

In the group of 195 patients evaluated, 446 operations had been performed. Complete clefts of lip and palate (CLP) were present in 99 patients (Veau III: *n* = 65, Veau IV: *n* = 34, 73 male, 26 female). 350 operations were performed for cleft repair in these patients. Isolated palatal clefts (CP) were present in 96 patients (Veau I: *n* = 11, Veau II: *n* = 85, 43 male, 53 female, 2 of Veau I palatal clefts were submucous clefts) with a total of 96 surgical procedures in this group of patients (Table 1).

**Table 1:** Patients categorized according to cleft type and follow-up interval

**Cleft type**	**Veau cat**	**Interval ≤ 6 years**	**Interval 7 – 14 years**	**Interval 15 – 22 years**	**Secondary surgery for ONF**	**Secondary surgery for VPI**	**Skeletal Surgery**
CLP	65 (III)	28	17	20			1
	34 (IV)	5	18	11	1		1
CP	10 (I)		6	4		1	
	86 (II)	29	30	27			
Total		62	71	62	1	1	2

The mean age at the time of operation was 5.7 months at lip closure, 11.7 months at hard palate repair and 14.3 months at soft palate closure. Cross-sectional analysis included data of 95 patients at follow-up intervals ≤ 6 years, data of 94 patients at intervals between 7—14 years, data of 85 patients at intervals between 15—22 years (Table 1).

### Secondary surgery

#### Oronasal fistulas

Two oronasal fistulas were registered: one ONF occurred in a patient with a bilateral cleft lip and palate within 4 weeks after soft palate repair at the transition between the hard and soft palate. Minor functional relevance resulted in repair surgery after 6 years and 2 months with uneventful healing. The second ONF occurred in a bilateral CLP patient 6 years after cleft repair during orthodontic palatal expansion. It was noted because negative intraoral pressure led to an appreciable sound of air flow from the nose into the oral cavity. No surgery was necessary as the perforation could not be sounded and was asymptomatic with no fluid / food regurgitation or loss of air through the nose or any other negative effects on speech quality. Secondary surgery for ONF was thus required in 1 out of 195 patients with cleft palate repair (0,5%). Due to the low frequency of surgery for ONFs, no statistical tests were possible.

#### Velopharyngeal insufficiency

Velopharyngeal corrective surgery was indicated in 1 patient (0.5%) secondary to the repair of a submucosal cleft of the soft palate due to insufficient muscle repair during primary surgery. In this case, a cranially pedicled mucosa-muscle flap from the dorsal pharyngeal wall was sutured to the soft palate (Sanvenero-Roselli) to narrow the oropharyngeal outlet 44 months after primary repair. As with the rate of surgery for ONFs, statistical tests were not possible due to the low frequency of VPI.

#### Skeletal corrective surgery

Secondary surgery for correction of cleft related skeletal deformities became necessary in 2 patients at the age of 18. In both patients, severe maxillary hypoplasia with midline deviation had been corrected using bimaxillary surgery with maxillary advancement / rotation and mandibular retrusion. Additionally, there were foreseeable cleft related skeletal Class III deformities refractory to orthodontic therapy in 4 patients in the age group of 15 – 17 years with scheduled skeletal correction after termination of facial growth. All these patients had a complete cleft lip and palate. Thus, as surgically induced maxillary growth retardation would be relevant and likely to occur mostly in patients with significant involvement of maxillary bone and nasal septum during dissection for cleft repair, only patients with complete unilateral and bilateral CLP in the age group of 15 – 22 years were considered as reference group (*n* = 31) resulting in a rate of 19.3% of secondary surgery for skeletal corrections (Table 1).

## Discussion

The present study evaluates a surgical cleft lip and palate treatment protocol that uses a two-step palatal repair with anterior to posterior closure for the ability to securely achieve the aims of the reconstructive concept: complete watertight palate closure and adequate velopharyngeal function. As a cross-sectional analysis of a single center experience, it represents not only the results of surgical concept but also the specific characteristics of the clinical interdisciplinary setting, which may limit its generalizability to other settings. Several reports have looked at the influence of timing, sequence of procedures and different techniques for palate closure on the occurrence of fistulae with contradictory results. Studies comparing 1 stage vs. 2 stage closure or different techniques for palate repair have reported a significant effect on oronasal fistula rates [21, 22] whereas others have not shown a significant influence of surgical techniques or sequence of surgical procedures [1, 2, 15, 23, 24]. The reported fistula rates ranged between 14.0 and 41.7%, but there were also much lower fistula rates of 5% and less reported for different protocols [11, 18, 19, 25].

An even more frequent reason for secondary palatal surgery is the correction of VPI. Systematic reviews that have looked at the rate of surgery for VPI have found higher rates of surgical interventions for VPI correction than those that had evaluated the rate of surgery for ONF repair [13, 14, 17]. This is paralleled by individual studies looking at the combined rate of surgical corrections of ONFs and VPI in their cohorts. In these reports, the rate of secondary surgery had been found to range from 12.8% to 17.2% for ONF repair and from 13.3% (VPI) to 25.9% for VPI correction [15, 16].

The present study has evaluated a comparably large cohort of CLP / CP patients and found a much lower rate of 0,5% of secondary surgery for both ONF and VPI correction. One of the reasons for the substantially lower rate of secondary interventions may be the anterior to posterior sequence of interventions that allowed for a controlled successive closure of individual cleft areas. The typical locations indicated in the Pittsburgh classification represent critical transition points between different cleft sections: alveolar cleft to primary palate cleft, primary palate cleft to secondary hard palate and secondary hard palate to soft palate. A sequence of surgical measures that moves from anterior to posterior may help to achieve a controlled successive closure at these critical points by allowing for easier mobilization of the soft tissues when the posterior cleft parts have not yet been closed. A safe buccal closure of the alveolar cleft during lip repair allows for a controlled closure of the palatal side of the alveolar cleft and the primary palate with the vomer flap that is extended to the mucoperiosteum of the premaxilla. The extension of the vomer flap to the premaxilla at the same time achieves secure closure of the critical transition between the primary palate and the secondary hard palate at the incisor foramen. This assumption is indirectly supported by the results of the protocols using vomer flaps for early hard palate closure at the time of lip repair in CLP patients showing very low rates of ONF [11, 18, 19, 25]. Finally, the securely closed hard palate provides a stable basis for the elongation of the palatal mucosa during closure of the soft palate and palatal muscle repair through the push back maneuver, resulting in a very low rate of secondary surgery for VPI correction. The extension of the incision to the mucoperiosteum of the premaxilla is limited in bilateral cases of CLP and may led to a shortage of mobile soft tissues to cover the cleft at the transition between primary palate cleft and (secondary) hard palate cleft. This may explain why both ONFs in the present study had occurred in patients with bilateral CLP.

The comparison of protocols based on the rate of ONF is nevertheless difficult, as there is no uniform inclusion of fistulae into the evaluation. A number of reports have considered only residual oronasal communications in the area of the hard and soft palate (Class I through IV of the Pittsburgh classification) without including ONFs at the incisor foramen (Class V) or the primary palate (Class VI and VII) [15, 26]. However, residual ONFs in the area of the incisor foramen are particularly difficult to close due to the lack of mobile and vascularized soft tissue that often makes a secure two-layered closure impossible. The present study has extended the claim of cleft palate repair to secure closure along the whole distance of the cleft from the alveolus to the uvula, as a significant number of Class V fistulae have shown to be in connection with fistulae of the alveolar cleft (Class VI and VII) [16]. This can substantially impair alveolar bone grafting for reconstruction of the alveolar crest at the time of canine eruption because safe coverage of the grafted bone in conjunction with fistula repair is challenging and may be difficult to achieve.

In order to achieve a secure repair of the primary palate cleft and the palatal part of the cleft alveolus, the concept of the present study has used a more extended dissection of palatal soft tissues in the area of the premaxilla. In conjunction with the mobilization of the vomerine mucosa this means a rather extensive subperiosteal mobilization of palatal and vomerine soft tissues in cleft repair of complete CLP. Extensive subperiosteal dissection of soft tissues in the area of the hard palate, the vomer and the premaxilla has been considered to be deleterious for midfacial growth and has been advised against [5]. However, more recent reports on the effect of surgical interventions for CLP repair suggest that it is not the surgery for palatal cleft repair but rather the timing and surgical procedure of lip repair that affect facial growth and the need for skeletal secondary surgery [6–8]). If we consider the rate of secondary skeletal corrective surgery as a proxy to the extent of surgically induced growth retardation, the presented concept is associated with a relatively low rate of skeletal corrections when compared to the previously reported rates of skeletal surgery in CLP patients between 40.0 and 54.8% [3, 27, 28]. It is clear that the incidence of orthognatic surgery in cleft patients is not a precise outcome measure for skeletal growth, which would require skeletal measurements or cephalometric evaluations. Therefore, direct conclusions on skeletal growth cannot be drawn. Nevertheless, the rate of skeletal corrective surgery has been previously used as an outcome parameter after surgical cleft repair with [27–29] or without the use of cephalometric data [8, 30]. Thus, it may be considered a quite commonly used approach to outcome assessment and a useful measure for comparison among different cohorts. In this way, the present results indicate that more extensive soft tissue dissection during the two steps of palatal cleft closure does not necessarily result in an increased need for skeletal surgery after completion of midfacial growth.

## Conclusions

The present study has shown that two-step sequential cleft palate closure of primary palate and hard palate first followed by soft palate closure has been associated with a minimal need of secondary corrective surgery for ONF and VPI at a relatively low rate of surgical skeletal corrections.

## Data Availability

Contact corresponding author.
